# Influence of Nodal signalling on pluripotency factor expression, tumour cell proliferation and cisplatin-sensitivity in testicular germ cell tumours

**DOI:** 10.1186/s12885-020-06820-6

**Published:** 2020-04-23

**Authors:** K. Harpelunde Poulsen, J. E. Nielsen, B. Grønkær Toft, U. N. Joensen, L. J. Rasmussen, M. Blomberg Jensen, R. T. Mitchell, A. Juul, E. Rajpert-De Meyts, A. Jørgensen

**Affiliations:** 1 Department of Growth and Reproduction, Copenhagen University Hospital (Rigshospitalet), Blegdamsvej 9, DK-2100 Copenhagen, Denmark; 2 International Research and Research Training Centre in Endocrine Disruption of Male Reproduction and Child Health (EDMaRC), Blegdamsvej 9, Copenhagen, Denmark; 3 Pathology Department, Copenhagen University Hospital (Rigshospitalet), Blegdamsvej 9, 2100 Copenhagen, Denmark; 4 Department of Urology, Copenhagen University Hospital (Rigshospitalet), Blegdamsvej 9, 2100 Copenhagen, Denmark; 5 Department of Cellular and Molecular Medicine, Center for Healthy Aging, University of Copenhagen, Blegdamsvej 3B, 2200 Copenhagen, Denmark; 6 MRC Centre for Reproductive Health, The Queen’s Medical Research Institute, University of Edinburgh, 47 Little France Crescent, Edinburgh, EH16 4TJ UK

**Keywords:** Nodal and Activin signalling, Expression of pluripotency factors, GCNIS, Testicular cancer, Cisplatin-sensitivity

## Abstract

**Background:**

Testicular germ cell tumours (TGCTs) are characterised by an overall high cisplatin-sensitivity which has been linked to their continued expression of pluripotency factors. Recently, the Nodal signalling pathway has been implicated in the regulation of pluripotency factor expression in fetal germ cells, and the pathway could therefore also be involved in regulating expression of pluripotency factors in malignant germ cells, and hence cisplatin-sensitivity in TGCTs.

**Methods:**

We used in vitro culture of the TGCT-derived cell line NTera2, ex vivo tissue culture of primary TGCT specimens and xenografting of NTera2 cells into nude mice in order to investigate the consequences of manipulating Nodal and Activin signalling on pluripotency factor expression, apoptosis, proliferation and cisplatin-sensitivity.

**Results:**

The Nodal signalling factors were markedly expressed concomitantly with the pluripotency factor OCT4 in GCNIS cells, seminomas and embryonal carcinomas. Despite this, inhibition of Nodal and Activin signalling either alone or simultaneously did not affect proliferation or apoptosis in malignant germ cells in vitro or ex vivo. Interestingly, inhibition of Nodal signalling in vitro reduced the expression of pluripotency factors and Nodal pathway genes, while stimulation of the pathway increased their expression. However, cisplatin-sensitivity was not affected following pharmacological inhibition of Nodal/Activin signalling or siRNA-mediated knockdown of the obligate co-receptor *CRIPTO* in NTera2 cells in vitro or in a xenograft model.

**Conclusion:**

Our findings suggest that the Nodal signalling pathway may be involved in regulating pluripotency factor expression in malignant germ cells, but manipulation of the pathway does not appear to affect cisplatin-sensitivity or tumour cell proliferation.

## Background

Testicular germ cell tumours (TGCTs) in young adults originate from a common precursor, germ cell neoplasia in situ (GCNIS, previously known as carcinoma in situ) [1–3]. GCNIS cells are considered to be transformed gonocytes that have failed to differentiate to pre-spermatogonia during fetal testis development, most likely as a consequence of altered signalling from the somatic niche [3]. The arrest of gonocyte maturation is regarded as the initial step in the testicular cancer pathogenesis and results in the presence of a sub-population of cells with retained expression of pluripotency factors in postnatal life [1–3]. Around puberty, the hormonal changes and re-organisation of the testes required to support spermatogenesis are hypothesised to promote increased proliferation and gain of invasive capacity of the GCNIS cells, resulting subsequently in formation of TGCTs. The two main types of TGCTs are seminoma (SEM) and non-seminoma (non-SEM), where the latter may contain undifferentiated embryonal carcinoma (EC) as well as the more differentiated yolk sac tumour (YST), choriocarcinoma (CHC) and teratoma (TER) components [4].

Treatment of TGCTs depends on the tumour subtype and stage, with generally excellent cure rates even for advanced disease. In most cases, orchiectomy followed by surveillance is sufficient, but TGCTs that metastasise (most often non-SEMs) may require a combination of surgery, chemotherapy and in a few cases radiation therapy [5]. TGCTs are generally highly sensitive to cisplatin-based chemotherapy presumably due to their fetal germ cell origin, but the mechanisms underlying this overall high cisplatin-sensitivity of TGCTs and occasional treatment resistance, are not understood in detail [6, 7]. An association between the embryonic phenotype of the majority of TGCTs and high cisplatin-sensitivity has been established, whereas the differentiated types of TGCTs, such as TER and CHC, are generally considered more resistant to treatment [8]. The relationship between expression of pluripotency factors and cisplatin-sensitivity has also been examined in a TGCT-derived EC cell line, in which loss of pluripotency factor expression by siRNA-mediated knockdown of *OCT4*, resulted in decreased sensitivity to cisplatin [9]. Moreover, treatment with retinoic acid to induce differentiation of the EC-derived NTera2 cell line along the neuroectodermal lineage, resulted in decreased expression of the pluripotency factor OCT4 and increased cisplatin-resistance [10–12], thus supporting the association between pluripotency factor expression and cisplatin-sensitivity. Although cisplatin-based chemotherapy has provided high cure rates for TGCTs, the treatment regimen is associated with long-term complications, including cardiovascular side effects and infertility as well as relapse [5, 13]. Therefore, optimisation of the current treatment regimen would be beneficial.

Despite the general understanding that expression of pluripotency factors is a hallmark of GCNIS and the majority of TGCTs, the underlying molecular mechanisms responsible for the maintenance or re-activation of pluripotency factor expression in these malignant germ cells are not well understood. Several independent studies have implicated the Nodal signalling pathway in the pathogenesis of TGCTs [12, 14–19]. Recently, we found that stimulation of the Nodal pathway in human fetal testes prolonged the expression of OCT4 in gonocytes, thus directly implicating the pathway in the regulation of the gonocyte to pre-spermatogonia transition during human fetal testis development [19] and involvement in regulating pluripotency factor expression in fetal germ cells (reviewed in [20]). Furthermore, high expression of the Nodal signalling factors *NODAL*, *LEFTY1* and *CRIPTO* has been reported in GCNIS cells, TGCTs and TGCT-derived cell lines [12, 16, 17], and several studies have found co-expression of Nodal signalling and pluripotency factors in NTera2 cells [12, 15]. Also, heterogeneous expression of the co-receptor CRIPTO was found in NTera2 cells, with highest expression in the subpopulation of cells displaying the most tumorigenic potential [15].

Nodal and Activin signal through essentially the same receptors, including the activin receptors type 1 (Alk4/7) and type 2 (ActRIIA/IIB). An important difference is that Nodal also requires the presence of the co-receptor Cripto for signal transduction. Among the target genes of the Nodal pathway are *Nodal* itself and the endogenous inhibitor *Lefty1/2*, which blocks the formation of the receptor complex by binding Nodal directly or by interacting with Cripto [21]. The endogenous inhibitor of Activin signalling is Follistatin, which binds directly to Activin to inhibit this pathway. In recent years, Nodal signalling has emerged as a promising therapeutic target due to its aberrant re-expression and signalling in various types of cancers, including breast cancer, melanoma, prostate cancer and pancreatic cancer [22–26]. Interestingly, we found that simultaneous inhibition of Nodal and Activin signalling resulted in an almost complete loss of gonocytes in human fetal testes [19]. Despite the implication of the Nodal signalling pathway in the pathogenesis of TGCTs, the mechanisms by which this signalling pathway is dysregulated in TGCTs remain to be elucidated. Therefore, we hypothesised that dysregulation of the Nodal signalling pathway is involved in the regulation of pluripotency factor expression and proliferation in malignant germ cells, and thus is associated with the characteristically high cisplatin-sensitivity of these cells.

## Methods

The aim of the this study was to investigate the involvement of Nodal signalling in the regulation of pluripotency factor expression, tumour cell proliferation and cisplatin-sensitivity in malignant germ cells by experimental manipulation of this pathway in NTera2 cells in vitro and in a xenograft model as well as in primary ex vivo cultures of adult testis tissue containing GCNIS cells.

### Human tissue sample collection and preparation

Tissue samples used in this study were collected according to the Helsinki Declaration following approval by the regional ethics committee (H-1-2012-007) and all patients gave their informed written and oral consent prior to surgery. Testicular tissue with and without presence of GCNIS cells and testicular tumour samples were obtained after orchiectomy for testicular cancer. Tissue samples were transported to the Pathology Department (Copenhagen University Hospital) immediately after the orchiectomy where a pathologist examined the testes, dividing it into tumour and macroscopically normal areas. The majority of the tissue was used for diagnostic evaluation, while the remaining tissue was allocated to research and either snap-frozen and stored at -80 °C or fixed in formalin or Bouin’s fixative. Alternatively, the collected testis tissues were placed in cell culture media, immediately transported to the laboratory and set up in ex vivo culture as described below. Testis specimens included samples of ‘normal testis (NT)’, samples containing GCNIS, SEM, EC and TER (only used for gene expression analysis). Tissue fragments with normal morphology and containing complete spermatogenesis without the presence of malignant germ cells were used as ‘normal adult testis’ controls. All tissue samples were evaluated by an experienced pathologist using a panel of immunohistochemical markers to characterise GCNIS cells and tumour subtypes, including placental-like alkaline phosphatase (PLAP), podoplanin (PDPN/D2–40), OCT4 (POU5F1), and (for non-SEMs only) also alpha-fetoprotein (AFP) and beta-choriogonadotropin (hCG) [27]. Frozen tissue specimens used for gene expression analysis were sectioned from each end of the tissue fragment and evaluated using immunohistochemistry to confirm the histological tumour subtype prior to RNA extraction.

### Immunohistochemistry

Immunohistochemistry on formalin-fixed tissue was performed as previously described in detail [28]. The fixed tissue samples were dehydrated, paraffin-embedded and sectioned (4 μm). Immunohistochemistry on Bouin’s fluid-fixed testicular tissue, TGCT samples and formalin-fixed NTera2 cells grown on glass slides was conducted using a pressure cooker for antigen-retrieval as previously described [19]. Visualisation was performed with ImmPACT DAB peroxidase substrate (Vector Laboratories, Burlingame, CA, US). Primary antibodies, dilutions and retrieval buffers are listed in Table 1. All sections were counterstained with Mayer’s haematoxylin before mounting with Aquatex (Merck, Kenilworth, NJ, US). Positive and negative controls were included for both protocols. Positive control samples included tissue/cells known to express the studied protein, including OCT4 (adult testes with GCNIS cells), NODAL, CRIPTO, LEFTY (mouse fetal testes and EC tumours), cPARP (adult testes, nuclease-treated) and BrdU (fetal testis culture, BrdU-treated). Negative controls were processed with the primary antibody replaced by the dilution buffer alone with none of the negative controls exhibiting any staining. Sections were evaluated using a Nikon Microphot-FXA microscope, subsequently scanned using a Nano-Zoomer 2.0 HT (Hamamatsu Photonics, Herrsching am Ammersee, Germany) and analysed using the software NDPview version 1.2.36 (Hamamatsu Photonics).

**Table 1:** Antibody dilutions, retrieval buffer and details. Antigen-retrieval buffers: citrate buffer, 10 mM, pH 6.0; TEG buffer, 10 mM Tris, 0.5 mM EGTA, pH 9.0

Antibody	Dilution	Retrieval buffer	Species	Company	Cat. Number
OCT4	1:50	TEG	Mouse	Santa Cruz	Sc-5279
NODAL	1:800	Citrate	Mouse	Abcam	Ab55676
CRIPTO	1:200	Citrate	Rabbit	Abcam	Ab19917
LEFTY	1:4000	Citrate	Rabbit	Abcam	Ab22569
cPARP	1:100	Citrate	Rabbit	Cell Signaling	5625
BrdU	1:100	Citrate	Mouse	Dako	M0744

### Quantitative RT-PCR

Quantitative RT-PCR (RT-qPCR) was performed as previously described [19] using the QuantStudio 3 Real-Time PCR System (Thermo Fisher, Rochester, NY, US). In brief, mRNA was extracted from frozen tissue specimens and cell lines using the RNAqueous Micro Kit (Ambion, Austin, TX, US) or Nucleospin RNA purification kit (Macherey-Nagel, Düren, Germany), respectively. cDNA was synthesised using 1 μg mRNA, a dT20 primer and random hexamers, resulting in 100 μl cDNA, with 1 μl cDNA used for each RT-qPCR reaction. Gene expression was analysed using specific primers (Table 2) designed to span intron-exon boundaries. All primers had previously been verified (Eurofins Genomics, Ebersberg, Germany). cDNA used for gene expression analysis in this study have also been used in a previous study [29]. RT-qPCR analyses were measured as duplicates and triplicates for frozen tissue specimens and cell line RNA extracts, respectively, using Brilliant II SYBR Green qPCR Master mix (Aligent technologies, Santa Clare, CA, US). The thermal cycling programme was: 95 °C for 15 min followed 40 cycles of 95 °C for 15 s and 62 °C for 1 min. Changes in gene expression were examined using the 2^-∆∆^Ct method [30]. Expression levels were normalised to *RPS20* or *RPS29* and calculated as a ratio with NT samples or vehicle controls set to 1.

**Table 2:** Primer sequences

Gene	Forward primer 5′-3′	Reverse primer 5′-3′	Amplicon size	GenBank Accession no.
*OCT4 (POU5F1)*	TACTCCTCGGTCCCTTTCC	CAAAAACCCTGGCACAAACT	166 bp	NM_002701
*NANOG*	TGATTTGTGGGCCTGAAGAAAA	GAGGCATCTCAGCAGAAGACA	60 bp	NM_024865.4
*NODAL*	AGCATGGTTTTGGAGGTGAC	CCTGCGAGAGGTTGGAGTAG	160 bp	NM_001329906.1
*CRIPTO*	TCCTTCTACGGACGGAACTG	ATCACAGCCGGGTAGAAATG	153 bp	NM_001174136.1
*LEFTY1*	GCCTCGACAGTGCATCGCCTC	CAAGTAAACAATGACACATTGGGC	477 bp	NM_020997.4
*RPS20*	AGACTTTGAGAATCACTACAAGA	ATCTGCAATGGTGACTTCCAC	179 bp	NM_001023
*RPS29*	CGCTCTTGTCGTGTCTGTTCA	CCTTCGCGTACTGACGGAAA	91 bp	NM_001032

### Culture of TGCT-derived NTera2 cell line

The TGCT-derived embryonal carcinoma cell line NTera2 was a kind gift from Professor Peter Andrews (University of Sheffield, UK) [31]. The NTera2 cells were cultured according to standard culture conditions. In brief, cells were cultured in DMEM supplemented with 10% fetal bovine serum, glutamine (58.5 mg/ml), penicillin (100 U/ml) and streptomycin (100 mg/ml) at 37 °C in a 5% CO_2_ atmosphere. Cell media and reagents were from Gibco (Invitrogen, Carlsbad, CA, US). For gene expression analyses and co-treatment experiments, the ALK4/5/7 inhibitor SB431542 [32] that simultaneously inhibits Nodal and Activin signalling (40 μM, 20 μM, 10 μM, 4 μM), recombinant Nodal (50 ng/ml), recombinant Activin (50 ng/ml), recombinant Lefty (100 ng/ml), recombinant Follistatin (100 ng/ml) and cisplatin (1 and 5 μM, stock solution 1 mg/ml dissolved in 0.9% NaCl from EberwePharma, Unterach am Attersee, Austria) were added to the media. SB431542 was dissolved in DMSO, while Nodal, Activin, Lefty and Follistatin were dissolved in PBS with 0.1% BSA or 4 mM HCl, 0.1% BSA in PBS. The recombinant proteins were purchased from R&D systems (Minneapolis, MN, US), while SB431542 was purchased from Sigma Aldrich (St. Louis, MO, US). Cells were plated in 25 cm^2^ flasks (Nunc, Thermo Fisher), incubated overnight before treatment was initiated and during the treatment period cells were split every 48 h with complete media change. Half of the cells were plated in new 25 cm^2^ flasks and the other half were collected for analysis. NTera2 cells used for immunohistochemical analysis were grown on glass slides (Nunc™ Lab-Tek™ II Chamber Slide™ System) for 48 h followed by fixation in 4% formalin. Slides were stored at 4 °C until further analysis.

### Cell proliferation assay

Proliferation of NTera2 cells was determined after 24 h and 48 h treatment with SB431542, and after co-treatments with SB431542 or recombinant Lefty and cisplatin. Proliferation was evaluated using the WST-1 assay according to the manufacturer’s instructions (Roche, Basel, Schweiz). 10,000 NTera2 cells were seeded into a 96-well plate with sixteen replicates of each sample and incubated overnight. Cells were then treated with SB431542 (5 μM, 10 μM and 20 μM) or vehicle control (0.1% DMSO) for 24 h and 48 h. To assess proliferation, 1:10 WST-1 dye (Roche) in serum-free DMEM was added to the cells for 2 h before absorbance was measured at 450 nm and 630 nm using a FLUOstar Omega microplate reader (BMG Labtech, Ortenberg, Germany) or an Epoch Microplate Spectrophotometer (Biotek, Brøndby, Denmark). For co-treatments, 4000 cells/well were plated into 96-well plates with eight replicates of each sample and allowed to attach for 6 h. Treatment with SB431542 (5 μM and 20 μM), recombinant Lefty (100 ng/ml) or vehicle control (0.1% DMSO) was then initiated for 48 h. Subsequently, media were removed and replaced with media containing cisplatin (1 μM and 5 μM) or 0.9% NaCl for 48 h. Cell proliferation was assessed with the WST-1 assay as described above.

### Ex vivo culture of adult human testis samples

Testis tissue samples (NT/GCNIS) obtained from orchiectomised testicular cancer patients (described above) were set up ex vivo in hanging drop cultures as described previously [33], with a few modifications. In brief, hanging drops were set up using 40 μl drops of culture medium ± treatment with addition of a single testicular fragment (1 mm^3^) per drop and complete media change every 48 h. Nine tissue fragments were set up for each treatment. Medium composition was: DMEM:F12, penicillin (100 U/ml), streptomycin (100 mg/ml), insulin, transferrin, selenium (× 1) and 10% fetal bovine serum. Media and supplements were all purchased from Gibco. Cultures were incubated for 48 h and 4 days at 34 °C in 5% CO_2_. 6 h before the end of the culture period, the testis pieces were incubated with BrdU-labelling reagent (Invitrogen). Subsequently, tissue fragments were fixed in 4% formalin.

### siRNA-mediated knockdown of *CRIPTO* expression

siRNA-mediated knockdown was carried out as previously described [12]. siRNA specific for *CRIPTO* (TDGF1-HSS144243, Invitrogen), a non-specific siRNA control (MISSION siRNA Universal Negative Control, SICOO1, Sigma Aldrich) and transfection agent RNAiMAX Lipofectamine (Life Technologies, Carlsbad, CA, US) was used. In brief, 1 × 10^6^ NTera2 cells were seeded into a 6-well plate and at the time of transfection cells were approximately 60–70% confluent. A concentration of 50 nM siRNA was used. 24 h after transfection, cells were re-plated into a 96-well plate (4000 cells/well) or cultured in T-25 cm^2^ flasks for RNA extractions. After 48 h, media were removed from the 96-well plate and replaced with media containing cisplatin (1 μM or 5 μM) or 0.9% NaCl for 48 h. Cell proliferation was determined by the WST-1 assay as described above.

### Establishment of NTera2 xenografts and treatments in NMRI nude mice

The establishment and experiments conducted in this model were set up by technicians at Pipeline Biotech A/S (Trige, Denmark). Animal experiments were conducted in compliance with the Danish Animal Experiments Inspectorate (license number 2011/561–1956) as previously described [10, 34], with few modifications. Briefly, 30 NMRI male mice (Fox^nu1^) aged 6–8 weeks (Janvier labs, Le Genest-Saint-Isle, France) were injected once with 2 × 10^6^ NTera2 cells into each flank. When the tumours reached an approximate size of 150 mm^3^, the mice were randomly allocated into three treatment groups of ten animals; treatment group 1, cisplatin (6 mg/kg i.p. once during experiment), treatment group 2, cisplatin + SB431542 (6 mg/kg cisplatin i.p. once during experiment and 10 mg/kg SB431542 i.p. 3 times weekly) and treatment group 3, vehicle (10 mg/kg DMSO i.p. 3 times weekly). Treatment groups 1 and 3 were also used in a separate study to reduce the total number of animals included (Lorenzen et al.*,* unpublished). Body weight and tumour volume were measured 3 times weekly throughout the experimental period of 14 days. Tumour volume was calculated as: tumour volume = length × width × ½ width. At the end of the experiment mice were euthanized by inhalation of CO_2_ followed by cervical dislocation. The mice were caged in European standard cages type II with Jeluxyl HW 300/500 bedding and the housing and changing system was designed to assure that MPF-status was preserved during the study. The air was exchanged approximately 12 times per hour and temperature was kept between 20 °C and 24 °C (controlled via the ambient ventilation system). Light cycle was 12-h dark and 12-h light. During the entire experimental period mice were fed ad libitum with Standard diet (Altromin 1234, 600 IE D3/kg diet; Altromin, Lage, Germany) and UV-sterilised water were administered ad libitum. All animals were inspected on a daily basis for their general condition. Any animal showing clinical signs of moderate pain or distress, any degree of suffering or clinical signs that exceed the limits of the study specific end-point would have been humanely euthanized according to the European and Danish legislation on animals in experimental studies.

### Statistical analysis

Statistical analysis was performed using the Software GraphPad Prism 8 (San Diego, CA, US). Differences in gene expression and cell proliferation were tested using a two-tailed Student’s t-test, while differences in tumour growth were tested using a one-way ANOVA with Bonferroni correction. Statistically significant differences are indicated as * *P* < 0.05, ** *P* < 0.01 and *** *P* < 0.001. The number of replicates in each experimental set-up and statistical significance are specified in figure legends.

## Results

### Expression of Nodal signalling factors in normal testis, GCNIS, testicular tumours and NTera2 cells

The expression levels of *NODAL*, *CRIPTO* and *LEFTY1* were initially investigated by RT-qPCR in tissue from adult testis samples with full spermatogenesis and no presence of malignant germ cells (hereafter termed ‘normal testis’ (NT)), samples containing pre-invasive GCNIS cells in the majority of tubules (GCNIS), seminoma tumour (SEM), embryonal carcinoma tumour (EC) and teratoma tumour (TER). *OCT4* (*POU5F1*) and *NANOG* were included to verify the neoplastic content in GCNIS, SEM and EC samples. Overall, the investigated Nodal pathway genes were all expressed in the included samples, but at very different levels (Supplementary Fig. 1a). In GCNIS, SEM and EC, the expression of *OCT4* (GCNIS, SEM, *P* < 0.05; EC, *P* < 0.001) and *NANOG* (all *P* < 0.05) was significantly increased compared to NT, verifying the neoplastic content within these samples. The expression of Nodal signalling factors *NODAL* (*P* < 0.001), *CRIPTO* (*P* < 0.05) and *LEFTY1* (*P* < 0.001) was significantly higher in EC samples compared to NT as well as GCNIS, SEM and TER.

Protein expression of NODAL, CRIPTO and LEFTY (antibody detects both LEFTY1 and LEFTY2) was examined by immunohistochemistry in serial sections of NT, GCNIS, SEM and EC. OCT4 was included as a marker of malignant germ cells, and was detected in GCNIS, SEM and EC, but not in NT which is in accordance with the expected expression pattern (Supplementary Fig. 1b). NODAL, CRIPTO and LEFTY were expressed in all investigated samples, except NT. The expression of all three Nodal pathway proteins was more pronounced in EC compared to GCNIS and SEM, with co-expression of all three proteins in OCT4^+^ EC cells. Interestingly, the expression of NODAL, CRIPTO and LEFTY was found only in a sub-population of GCNIS cells and expression in these cells was low. Additionally, the expression pattern of NODAL, CRIPTO, LEFTY and OCT4 was also examined in the EC-derived NTera2 cell line (Supplementary Fig. 1c). Noticeably, CRIPTO was strongly expressed in the nuclei in addition to the expected cytoplasmic/membranous expression. NODAL was expressed in the cytoplasm, whereas LEFTY appeared to be present between adjacent NTera2 cells, possibly reflecting secretion from the cells.

### Effects of simultaneous inhibition of Nodal and Activin signalling on proliferation and transcriptional expression in NTera2 cells

**Fig. 1:**
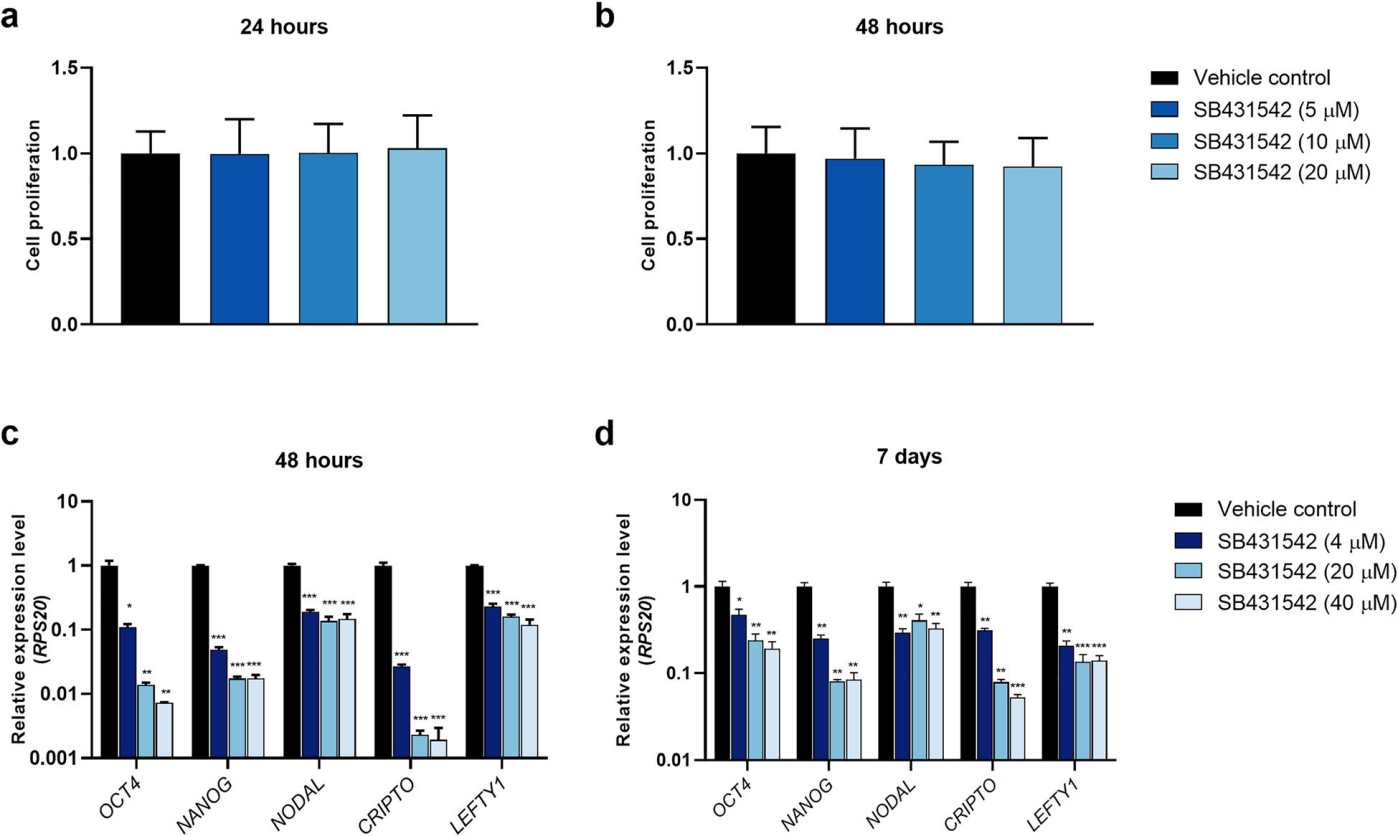
Effects of simultaneously inhibiting Nodal and Activin signalling in the NTera2 cell line. NTera2 cells were treated with 20 μM, 10 μM and 5 μM SB431542 for **a**) 24 h and **b**) 48 h followed by assessment of cell proliferation. Experiments were set up with 16 replicates per treatment and repeated in three independent experiments (*n* = 3). Cell proliferation is set to 1 in the vehicle controls. Expression of pluripotency factors (*OCT4*, *NANOG*) and Nodal pathway genes (*NODAL*, *CRIPTO*, *LEFTY1*) in NTera2 cells following **c**) 48 h and **d**) 7 days of treatment with SB431542 (40 μM, 20 μM and 4 μM). *RPS20* was used as reference gene. Expression level is set to 1 in the vehicle controls. Experiments were conducted in triplicates in three independent experiments (*n* = 3) and measured as technical triplicates. Values represent mean ± SEM. Significant difference compared to expression in vehicle control-treated cells, * *P* < 0.05, ** *P* < 0.01 and *** *P* < 0.001. Note logarithmic scale

In order to investigate the effects of inhibiting Nodal and Activin signalling on proliferation of NTera2 cells, treatment experiments with the ALK4/5/7 inhibitor SB431542 [32], were conducted. Treatment with 20 μM, 10 μM and 5 μM SB431542 had no significant effect (*P* > 0.05) on proliferation of NTera2 cells after 24 h or 48 h (Fig. 1a-b). In contrast, treatment with SB431542 (40 μM, 20 μM and 4 μM) for 48 h resulted in significantly lower expression of pluripotency factors *OCT4* (40 μM and 20 μM, *P* < 0.01, 4 μM, *P* < 0.05) and *NANOG* (*P* < 0.001) as well as *NODAL*, *CRIPTO* and *LEFTY1* (all *P* < 0.001) in NTera2 cells (Fig. 1c). After 7 days of treatment with SB431542 (40 μM, 20 μM and 4 μM), expression of all investigated genes remained significantly reduced (Fig. 1d).

### Effects of manipulating Nodal and Activin signalling separately on the transcriptional expression pattern in NTera2 cells and ex vivo cultures of adult testis tissue

**Fig. 2:**
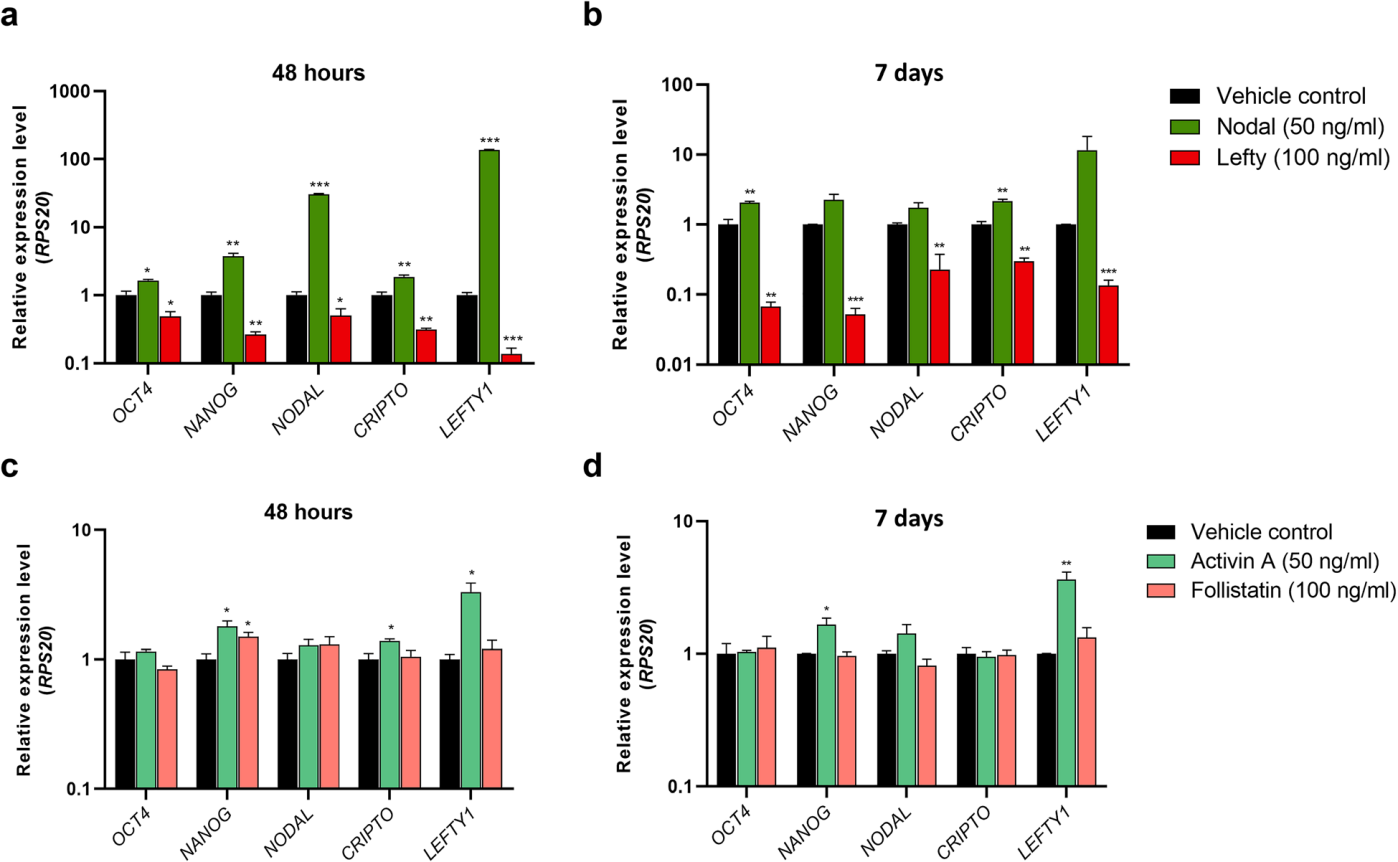
Effects of manipulating Nodal and Activin signalling separately in NTera2 cells. Expression of pluripotency factors (*OCT4*, *NANOG*) and Nodal signalling factors (*NODAL*, *CRIPTO*, *LEFTY1*) in NTera2 cells following **a**) 48 h and **b**) 7 days of treatment with recombinant Nodal (50 ng/ml) and Lefty (100 ng/ml) and **c**) 48 h and **d**) 7 days of treatment with Activin A (50 ng/ml) and Follistatin (100 ng/ml). *RPS20* was used as reference gene. Expression level is set to 1 in the vehicle controls. Experiments were conducted in triplicates in three independent experiments (n = 3) and measured as technical triplicates. Values represent mean ± SEM. Significant difference compared to expression in vehicle control-treated cells, * *P* < 0.05, ** *P* < 0.01 and *** *P* < 0.001. Note logarithmic scale

To separate the effects of inhibiting or stimulating Nodal and Activin signalling pathways individually, a series of treatment experiments were performed in the NTera2 cell line. The Nodal pathway was stimulated by treatment with recombinant Nodal (50 ng/ml) and inhibited by treatment with recombinant Lefty (100 ng/ml). Nodal treatment for 48 h resulted in a significant upregulation of *OCT4* (*P* < 0.05) and *NANOG* (*P* < 0.01) as well as *NODAL* (*P* < 0.001), *CRIPTO* (*P* < 0.01) and *LEFTY1* (*P* < 0.001) (Fig. 2a). The expression of *OCT4* and *CRIPTO* (both *P* < 0.01) remained upregulated after 7 days of treatment with recombinant Nodal (Fig. 2b). In contrast, Lefty treatment for 48 h significantly reduced the expression level of all investigated genes; *OCT4* (*P* < 0.05), *NODAL* (*P* < 0.05), *NANOG* (*P* < 0.01), *CRIPTO* (*P* < 0.01) and *LEFTY1* (*P* < 0.001) (Fig. 2a). The expression of *OCT4* (*P* < 0.01), *NANOG* (*P* < 0.001), *NODAL* (*P* < 0.01), *CRIPTO* (*P* < 0.01) and *LEFTY1* (*P* < 0.001) continued to be downregulated following Lefty treatment for 7 days (Fig. 2b). The Activin pathway was stimulated using recombinant Activin A (50 ng/ml) and inhibited using recombinant Follistatin (100 ng/ml). Activin treatment resulted in significantly higher expression of *NANOG* (*P* < 0.05) and *LEFTY1* (48 h, *P* < 0.05; 7 days, *P* < 0.01) at both 48 h and 7 days (Fig. 2c-d), with increased expression of *CRIPTO* (*P* < 0.05) also after 48 h. Follistatin treatment increased only the expression of *NANOG* (*P* < 0.05) after 48 h.

**Fig. 3:**
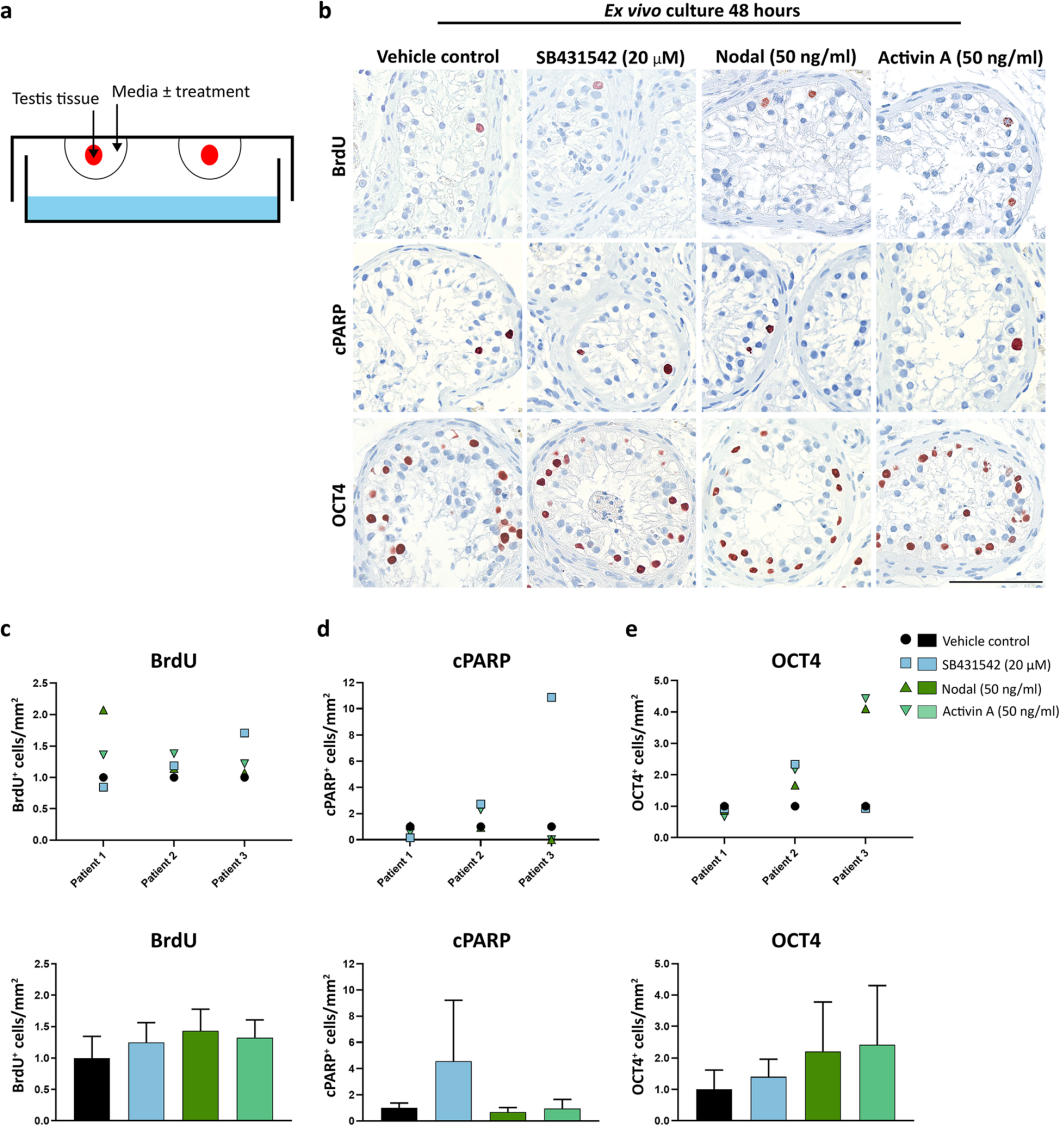
Effects of manipulating Nodal and Activin signalling in GCNIS cells cultured ex vivo for 48 h. **a**) Schematic illustration of the experimental ex vivo hanging drop culture approach. **b**) Immunohistochemical staining with BrdU (proliferation marker), cPARP (apoptosis marker) and OCT4 (pluripotency marker) in adult testis tissue containing GCNIS cells treated with SB431542 (20 μM), recombinant Nodal (50 ng/ml), Activin A (50 ng/ml) or vehicle for 48 h. Sections were counterstained with Mayer’s haematoxylin. Scale bar corresponds to 100 μm. Quantification of the number of **c**) BrdU^+^ cells per mm^2^, **d**) cPARP^+^ cells per mm^2^ and **e**) OCT4^+^ cells per mm^2^. Number of positive cells is normalised to vehicle controls (set to 1), and only apoptotic GCNIS cells were quantified. Tissue from three patients was evaluated (n = 3). Top panel of **c**-**e**) shows results from the individual patient samples, while bottom panel represents mean ± SEM

**Fig. 4:**
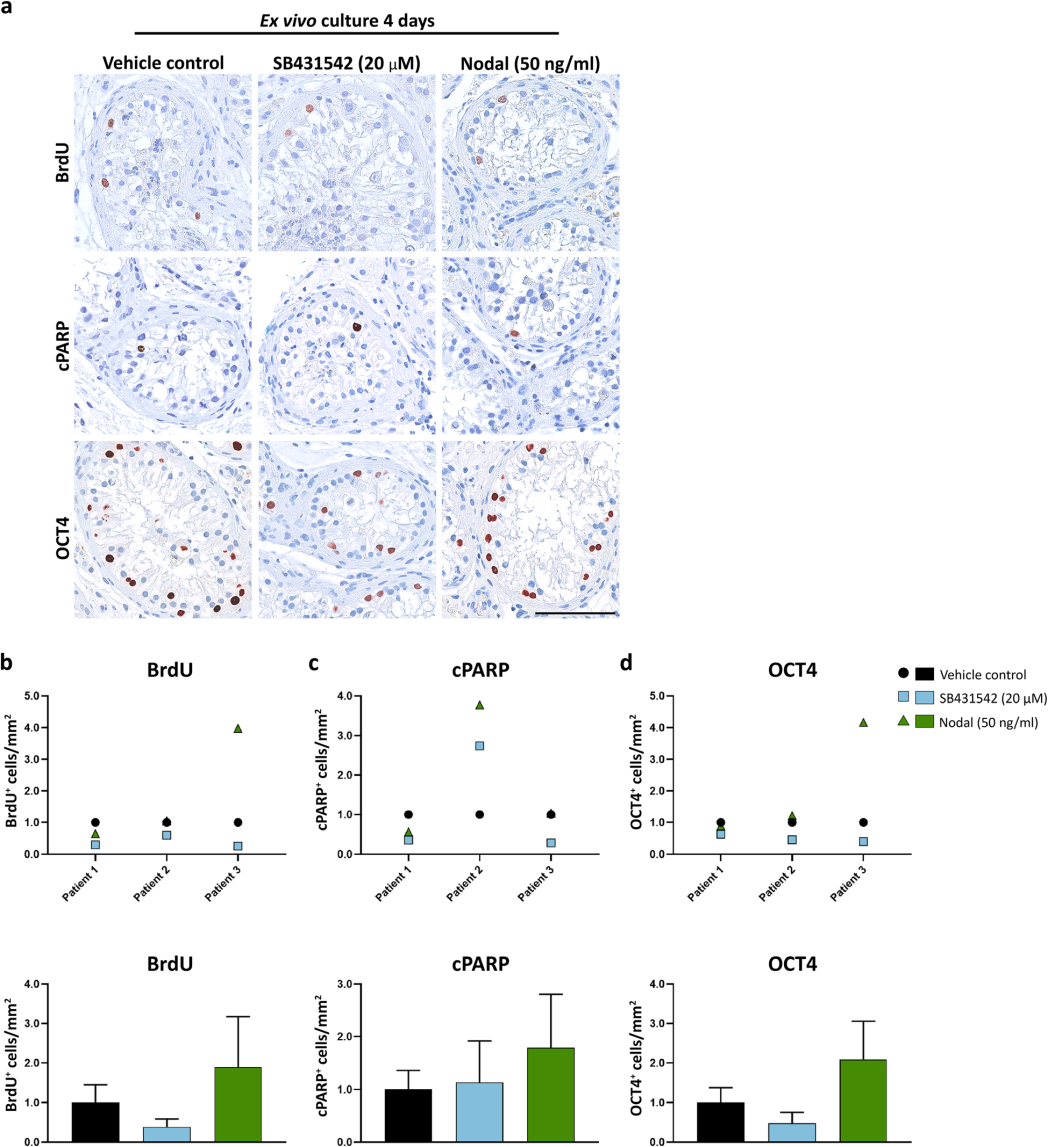
Effects of manipulating Nodal and Activin signalling in GCNIS cells cultured ex vivo for 4 days. **a**) Immunohistochemical staining with BrdU (proliferation marker), cPARP (apoptosis marker) and OCT4 (pluripotency marker) in adult testis tissue containing GCNIS cells treated with SB431542 (20 μM), recombinant Nodal (50 ng/ml) or vehicle for 4 days. Sections were counterstained with Mayer’s haematoxylin. Scale bar corresponds to 100 μm. Quantification of the number of **b**) BrdU^+^ cells per mm^2^, **c**) cPARP^+^ cells per mm^2^ and **d**) OCT4^+^ cells per mm^2^. Number of positive cells is normalised to vehicle controls (set to 1), and only apoptotic GCNIS cells were quantified. Tissue from three patients was evaluated (*n* = 3). Top panel of **b**-**d**) shows results from the individual patient samples, while bottom panel represents mean ± SEM

To examine the effects of manipulating Nodal and Activin signalling in malignant germ cells preserved within their somatic niche, the effects of SB431542, recombinant Nodal and Activin A were investigated in cultures of primary testis tissue from testicular cancer patients (Fig. 3 and Fig. 4). Tissue containing tubules with GCNIS cells (located adjacent to the TGCT tumour in the orchiectomised testis) from three patients was cultured ex vivo for 48 h (Fig. 3) and 4 days (Fig. 4). None of the treatments significantly affected (*P* > 0.05) proliferation (BrdU^+^/mm^2^), apoptosis (cPARP^+^/mm^2^) or number of GCNIS cells (OCT4^+^/mm^2^) compared to the vehicle controls after 48 h (Fig. 3b-e) or 4 days (Fig. 4a-d).

### Effects of pharmaceutical inhibition of Nodal and Activin signalling on cisplatin-sensitivity in NTera2 cells

**Fig. 5:**
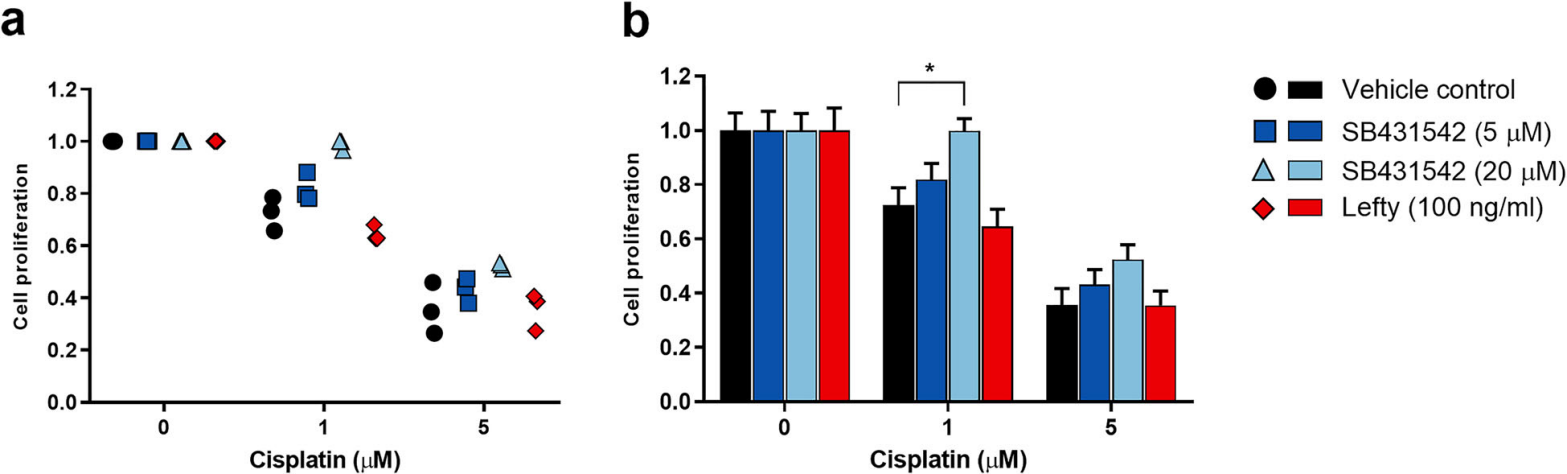
Effects of pharmaceutical inhibition of Nodal/Activin or Nodal signalling on cisplatin-sensitivity in NTera2 cells. NTera2 cells were treated for 48 h with SB431542 (5 μM and 20 μM) or Lefty (100 ng/ml) followed by additional 48 h of cisplatin treatment (1 μM and 5 μM) and assessment of cell proliferation. **a**) Experiments were conducted in three independent experiments with eight replicates per treatment (shown as mean for each experiment). Cell proliferation is set to 1 in NTera2 cells not treated with cisplatin. **b**) Values represent mean ± SEM from the three independent experiments (*n* = 3). Significant difference compared to proliferation of vehicle-treated control NTera2 cells receiving corresponding cisplatin treatment, * *P* < 0.05

To determine whether co-treatment with inhibitors of the Nodal signalling pathway affects cisplatin-sensitivity, NTera2 cells were treated with SB431542 (5 μM and 20 μM) or recombinant Lefty (100 ng/ml) for 48 h followed by 48 h of cisplatin treatment (1 μM and 5 μM). Subsequently, effects on cell proliferation was assessed. Co-treatments were conducted in three independent experiments, with similar results obtained between the experiments (Fig. 5a). Overall, initial exposure to SB431542 or Lefty followed by cisplatin treatment did not affect the cisplatin-sensitivity in the NTera2 cells. However, co-treatment with 20 μM SB431542 and 1 μM cisplatin significantly increased (*P* < 0.05) proliferation compared to cells treated with 1 μM cisplatin only, indicating that the cells were less sensitive to the cisplatin treatment (Fig. 5b). Generally, there was a tendency towards reduced cisplatin-sensitivity when cells were co-treated with SB431542 and cisplatin regardless of the doses used, although for most combinations of doses this was not statistically significant (*P* > 0.05).

### Effects of siRNA-mediated knockdown of *CRIPTO* expression on cisplatin-sensitivity in NTera2 cells

**Fig. 6:**
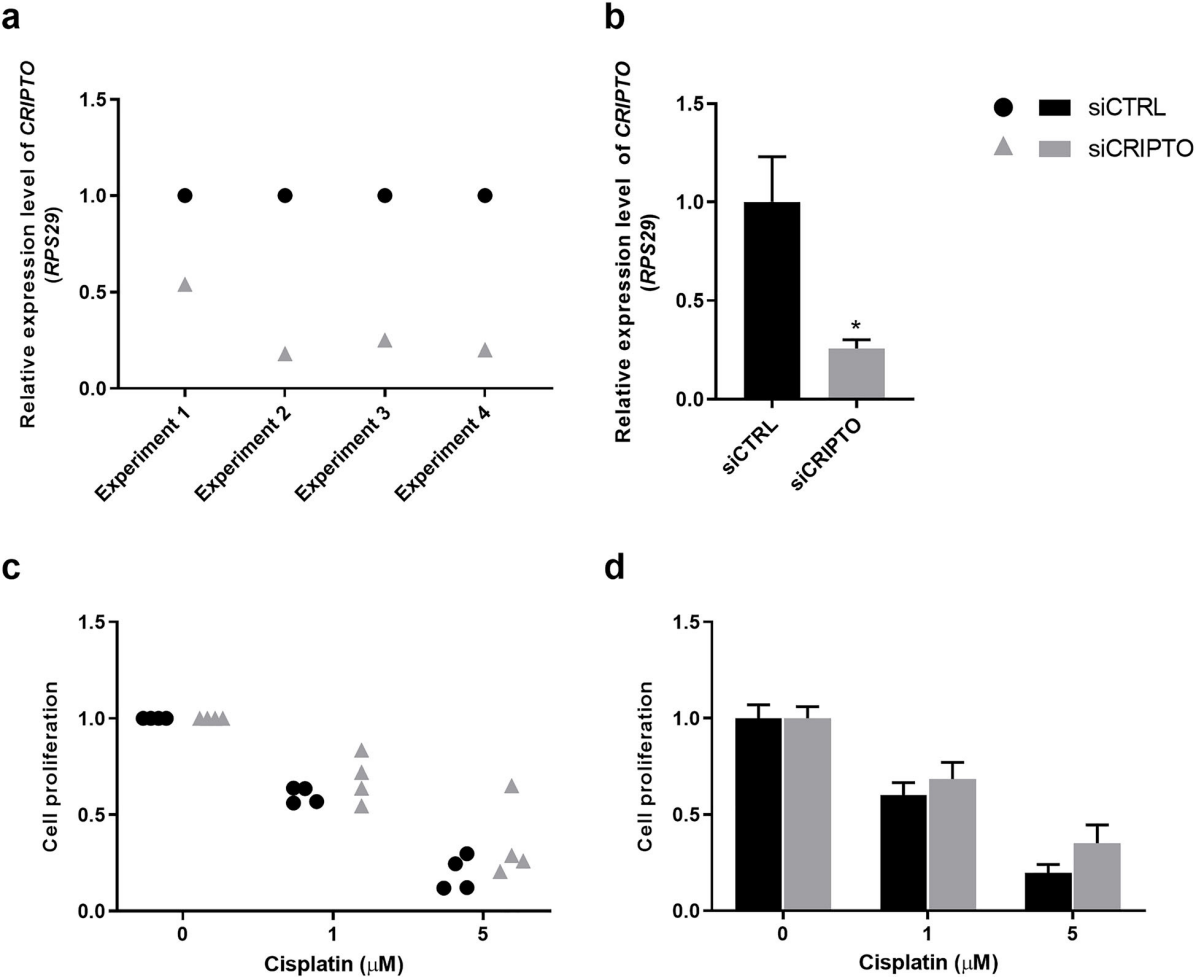
Effects of siRNA-mediated knockdown of *CRIPTO* expression on cisplatin-sensitivity in NTera2 cells. Expression of *CRIPTO* determined by RT-qPCR after knockdown using a siRNA targeting *CRIPTO* (siCRIPTO) and a non-specific siRNA control (siCTRL)*. RPS29* was used as reference gene. **a**) Experiments were conducted in four independent experiments (*n* = 4) and measured as technical triplicates (shown as mean for each experiment). Expression level of *CRIPTO* is set to 1 in vehicle-treated control NTera2 cells. **b**) Values represent mean ± SEM from the four independent experiments. Significant difference compared to expression level of *CRIPTO* in siCTRL-transfected NTera2 cells, * *P* < 0.05. Following knockdown, cells were subjected to treatment with 1 μM and 5 μM cisplatin for 48 h and cell proliferation was assessed. **c**) Experiments were conducted in four independent experiments and set up with eight technical replicates per treatment. **d**) Values represent mean ± SEM from the four independent experiments. Cell proliferation is set to 1 in vehicle-treated control NTera2 cells not treated with cisplatin

In order to examine whether the tendency towards reduced cisplatin-sensitivity found after inhibition of Nodal signalling (with SB431542) could be verified, the effect of *CRIPTO* knockdown on cisplatin-sensitivity was examined. Expression of the co-receptor *CRIPTO*, obligate for Nodal signalling, was knocked down in NTera2 cells by a siRNA approach. Subsequently, NTera2 cells were treated with cisplatin (1 μM and 5 μM) for 48 h and cell proliferation was assessed. The expression of *CRIPTO* was significantly reduced (*P* < 0.05) upon transfection with siCRIPTO compared to the siCTRL (Fig. 6a-b) in all individual experiments and when combined. Despite the significant reduction in *CRIPTO* expression (*P* < 0.05), no effect on cisplatin-sensitivity was found in the NTera2 cells when compared to the siCTRL-transfected NTera2 cells treated with cisplatin (Fig. 6c-d).

### Effects of simultaneous inhibition of Nodal and Activin signalling on cisplatin-sensitivity in a NTera2 xenograft mouse model

**Fig. 7:**
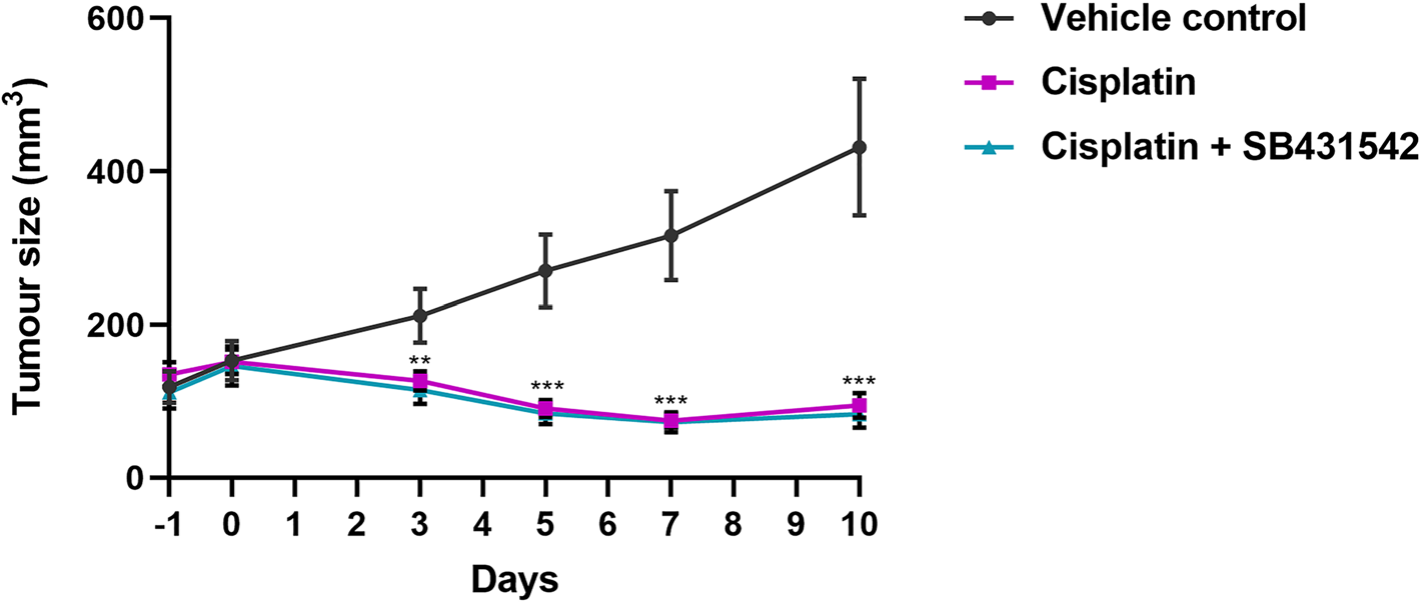
Effects of inhibiting Nodal/Activin signalling on cisplatin-sensitivity in a NTera2 xenograft model. NTera2 cells (2 × 10^6^; single injection) were xenografted into the flanks of nude mice and allowed to grow to an average size of 150 mm^3^. Animals were allocated into three groups (*n* = 10 for each group) and treated (day 0 on graph) with vehicle (10 mg/kg DMSO i.p. three times weekly), cisplatin (6 mg/kg i.p. once during the experiment) or cisplatin + SB431542 (6 mg/kg cisplatin i.p. once during the experiment and 10 mg/kg SB431542 i.p. three times weekly). Body weight and tumour size (mm^3^) were evaluated three times weekly during the experimental period of 11 days. Values represent mean ± SEM. Significant difference compared to vehicle control-treated mice, * *P* < 0.05, ** *P* < 0.01 and *** *P* < 0.001. Abbreviations: i.p., intraperitoneal injection

Simultaneous inhibition of Nodal and Activin signalling by SB431542 treatment in combination with cisplatin treatment was subsequently investigated in an NTera2 xenograft mouse model. NTera2 cells were injected into the flanks of nude mice and after the development of tumours, treatment was initiated. The animals were treated with either cisplatin alone, a combination of cisplatin + SB431542 or vehicle control. None of the animals exhibited observable negative effects of the experimental procedure or treatments. In the vehicle-treated control mice, tumour burden continued to increase throughout the experimental period (Fig. 7), while in mice treated with cisplatin alone and cisplatin + SB431542, the tumour size was significantly reduced (*P* < 0.01 and *P* < 0.001) compared to the vehicle controls already 3 days after the treatments were initiated. However, no significant difference (*P* > 0.05) in tumour size was found between mice treated with cisplatin alone and cisplatin + SB431542 at any of the evaluated time-point (Fig. 7).

## Discussion

In the present study, we found high expression of the Nodal signalling factors in undifferentiated types of TGCTs and our results suggest that the Nodal pathway may be involved in the regulation of pluripotency factor expression in malignant germ cells. However, targeting Nodal signalling in the EC-derived NTera2 cell line by several different experimental approaches did not affect proliferation of the malignant germ cells or result in any pronounced effects on cisplatin-sensitivity, indicating that the Nodal pathway may not represent a promising clinical target to augment the effect of current chemotherapy regimens in testicular cancer patients.

NODAL, CRIPTO and LEFTY1/2 were expressed at transcript and protein levels in GCNIS cells and the undifferentiated TGCTs, SEM and EC. The most pronounced expression of all Nodal pathway factors was found in EC and co-expressed in OCT4^+^ cells. In accordance with previous studies [14, 16, 18], the expression of Nodal factors in ‘normal’ testis without malignant cells was low/absent, suggesting maintenance of Nodal factor expression in GCNIS and/or re-activation in malignant germ cells. Additionally, the overall expression of the Nodal factors at both gene and protein levels in GCNIS and SEM was lower compared to EC which is in accordance with a previous study [18]. Interestingly, the protein expression of the Nodal pathway factors was less pronounced in GCNIS cells compared to EC but also to some extent SEM, which supports the notion that Nodal signalling components might be maintained only at low levels in GCNIS cells and are upregulated in the invasive tumours, although it remains to be determined whether the Nodal pathway is involved in the transition from GCNIS to EC/(SEM). The observed high expression of LEFTY1/2 in undifferentiated TGCTs, indicates that the inhibitory feedback mechanism on Nodal signalling may not be dysregulated in TGCTs, despite a previous study suggested this based on the reported low expression of *LEFTY1* in EC [16].

The Nodal signalling factors were all expressed in the NTera2 cell line, hence we used it as a model to examine effects of manipulating Nodal signalling in TGCTs. Simultaneous inhibition of Nodal and Activin pathways did not affect proliferation in NTera2 cells or in GCNIS cells in ex vivo cultured testis samples. Since Nodal and Activin signalling have been implicated in germ cell survival in both human and mouse fetal testes [19, 35], this may indicate a difference between normal fetal gonocytes and malignant germ cells, although it is important to consider that in contrast to normal fetal germ cells, malignant TGCTs have acquired features allowing them to survive outside of their normal niche. In the NTera2 cells, simultaneous inhibition of Nodal and Activin signalling resulted in reduced expression of the investigated pluripotency factors and Nodal pathway genes, and after separating the effects of Nodal and Activin signalling pathways it was evident that the Nodal pathway was responsible for the majority of observed effects. Additionally, the opposing effects observed after stimulating and inhibiting Nodal signalling, suggest that the pathway may be involved in the regulation of pluripotency factor expression in malignant germ cells. These results are in accordance with the reported involvement of Nodal signalling in regulating pluripotency factor expression in human fetal testes [19], mouse fetal testes [16, 36, 37] and human embryonic stem cells [38]. However, manipulation of Nodal and Activin signalling in ex vivo cultures of GCNIS-containing tissue (48 h and 4 days) did not overall affect the number of OCT4^+^ GCNIS cells, although in one patient sample an increased number of OCT4^+^ GCNIS cells was observed following treatment with Nodal and Activin (48 h) and Nodal (4 days). The different responses between patient samples as well as the relative short-term culture periods used in this study, suggest that additional studies examining the effects of stimulated Nodal/Activin signalling on GCNIS cells may be relevant. Importantly, the overall low number of apoptotic germ cells and the presence of proliferating cells in the cultured tissue samples, suggest that the tissue was supported by the culture approach and that the selected treatment doses were not toxic. However, given the high variation in the tissue, which is expected in testicular cancer patients [33], minor effects of treatments can be difficult to detect using this experimental approach.

Aberrant re-activation of Nodal signalling has been reported in various types of cancers, including cancer stem cells which also express pluripotency factors, and several studies have shown that inhibition of Nodal/(Activin) signalling reduces the tumorigenic potential both in vitro and in cancer mouse models [22–26]. Additionally, combined treatment with the SB431542 inhibitor and chemotherapy (gemcitabine) abolished tumours in a pancreatic cancer mouse model and resulted in complete survival of mice in this treatment group [24]. Moreover, given the almost complete germ cell loss following SB431542 treatment of human fetal testes, we examined whether co-treatment with this inhibitor could augment cisplatin-sensitivity in NTera2 cells. Overall, we did not observe pronounced effects on cisplatin-sensitivity following inhibition of Nodal signalling in vitro or in the xenograft model, except tendencies toward reduced cisplatin-sensitivity after in vitro pharmaceutical inhibition of Nodal/Activin signalling and siRNA-mediated knockdown of the obligate co-receptor *CRIPTO.* In vitro treatment with recombinant Lefty to inhibit only Nodal signalling did not result in reduced cisplatin-sensitivity (or showed tendencies in this direction), which could reflect the different levels at which inhibitory molecules and siRNA-mediated knockdown functions [39]. Additionally, the slightly increased cisplatin-resistance observed following blockage of both Nodal and Activin signalling (using the high dose of SB431542), but not after specific inhibition of Nodal signalling, could indicate some redundancy between the Nodal and Activin pathways. We speculate that inhibition of Nodal signalling promotes downregulation of pluripotency factor expression in the malignant germ cells driving them towards a more differentiated phenotype, which is associated with reduced cisplatin-sensitivity [8–12]. However, since both ECs (high Nodal expression) and SEMs (low Nodal expression) are highly sensitive to cisplatin-based treatment [6, 7], this may explain why we did not find pronounced effects on cisplatin-sensitivity upon manipulation of the Nodal signalling pathway. It has previously been demonstrated that calcitriol treatment (active form of vitamin D) of NTera2 cells resulted in both decreased expression of pluripotency factors and augmented effects of cisplatin, while treatment of NTera2 with retinoic acid decreased the expression of pluripotency factors and reduced cisplatin-mediated effects [10, 34]. This suggests that the relationship between pluripotency factor expression and cisplatin-sensitivity in malignant germ cells is not completely understood and that further studies examining this would be relevant.

## Conclusions

In conclusion, the Nodal signalling factors are highly expressed in the undifferentiated types of TGCTs and may be involved in the regulation of pluripotency factor expression in malignant germ cells. In contrast to several other types of cancers in which the Nodal pathway is also re-activated, inhibition of Nodal (and Activin) signalling did not affect tumour cell proliferation or augment cisplatin-sensitivity in TGCTs in vitro or in the xenograft model. Thus, the different response to the pharmaceutical pathway inhibitor SB431542 in TGCTs compared to human fetal germ cells, suggests that regulation or feedback mechanisms related to the Nodal pathway may be altered in malignant germ cells, although additional studies are needed to examine these mechanisms in more detail.

## Supplementary information

**Additional file 1: Figure S1.** Expression of Nodal signalling factors in testis, GCNIS cells, TGCTs and TGCT-derived NTera2 cells. a) Expression level of *OCT4*, *NANOG*, *NODAL*, *CRIPTO* and *LEFTY1* in testis tissue with complete spermatogenesis and no malignant germ cells (NT), testis samples containing GCNIS cells (GCNIS), seminoma tumour (SEM), embryonal carcinoma (EC) and teratoma (TER) examined by RT-qPCR. *RPS20* was used as reference gene. Expression level is set to 1 in NT samples. Tissue samples from eight patients were included (*n* = 8) and measured as technical duplicates. Values represent mean ± SEM. Significant difference compared to expression in NT samples, * *P* < 0.05, ** *P* < 0.01 and *** *P* < 0.001. Note logarithmic scale. b-c) Expression of Nodal signalling factors NODAL, CRIPTO and LEFTY (antibody detects both LEFTY1 and LEFTY2) determined by immunochemical analysis in serial sections of NT, GCNIS, SEM and EC and TGCT-derived NTera2 cells. OCT4 is included as a marker of malignant germ cells. Sections were counterstained with Mayer’s haematoxylin. Scale bars correspond to 50 μm (b) and 100 μm (c).

## Data Availability

The data to support the findings of this study are available upon reasonable request from the corresponding author, but restrictions apply to the availability of these data, which were used under license for the current study, and so are not publicly available.

## Abbreviations

CHC: Choriocarcinoma
EC: Embryonal carcinoma
GCNIS: Germ cell neoplasia in situ
i.p.: Intraperitoneal injection
Non-SEM: Non-seminoma
NT: Normal testis
SEM: Seminoma
TER: Teratoma
TGCTs: Testicular germ cell tumours
YST: Yolk sac tumour
