# Supplementary material for: Influence of Nodal signalling on pluripotency factor expression, tumour cell proliferation and cisplatin-sensitivity in testicular germ cell tumours
Source: BMC Cancer. 2020 Apr 23;20:349. doi: 10.1186/s12885-020-06820-6 (PMC7181506; doi:10.1186/s12885-020-06820-6)
Supplement: Supplementary file 1 — Additional file 1: Figure S1. Expression of Nodal signalling factors in testis, GCNIS cells, TGCTs and TGCT-derived NTera2 cells. a) Expression level of OCT4, NANOG, NODAL, CRIPTO and LEFTY1 in testis tissue with complete spermatogenesis and no malignant germ cells (NT), testis samples containing GCNIS cells (GCNIS), seminoma tumour (SEM), embryonal carcinoma (EC) and teratoma (TER) examined by RT-qPCR. RPS20 was used as reference gene. Expression level is set to 1 in NT samples. Tissue samples from eight patients were included (n = 8) and measured as technical duplicates. Values represent mean ± SEM. Significant difference compared to expression in NT samples, * P < 0.05, ** P < 0.01 and *** P < 0.001. Note logarithmic scale. b-c) Expression of Nodal signalling factors NODAL, CRIPTO and LEFTY (antibody detects both LEFTY1 and LEFTY2) determined by immunochemical analysis in serial sections of NT, GCNIS, SEM and EC and TGCT-derived NTera2 cells. OCT4 is included as a marker of malignant germ cells. Sections were counterstained with Mayer’s haematoxylin. Scale bars correspond to 50 μm (b) and 100 μm (c). [file 12885_2020_6820_MOESM1_ESM.docx]

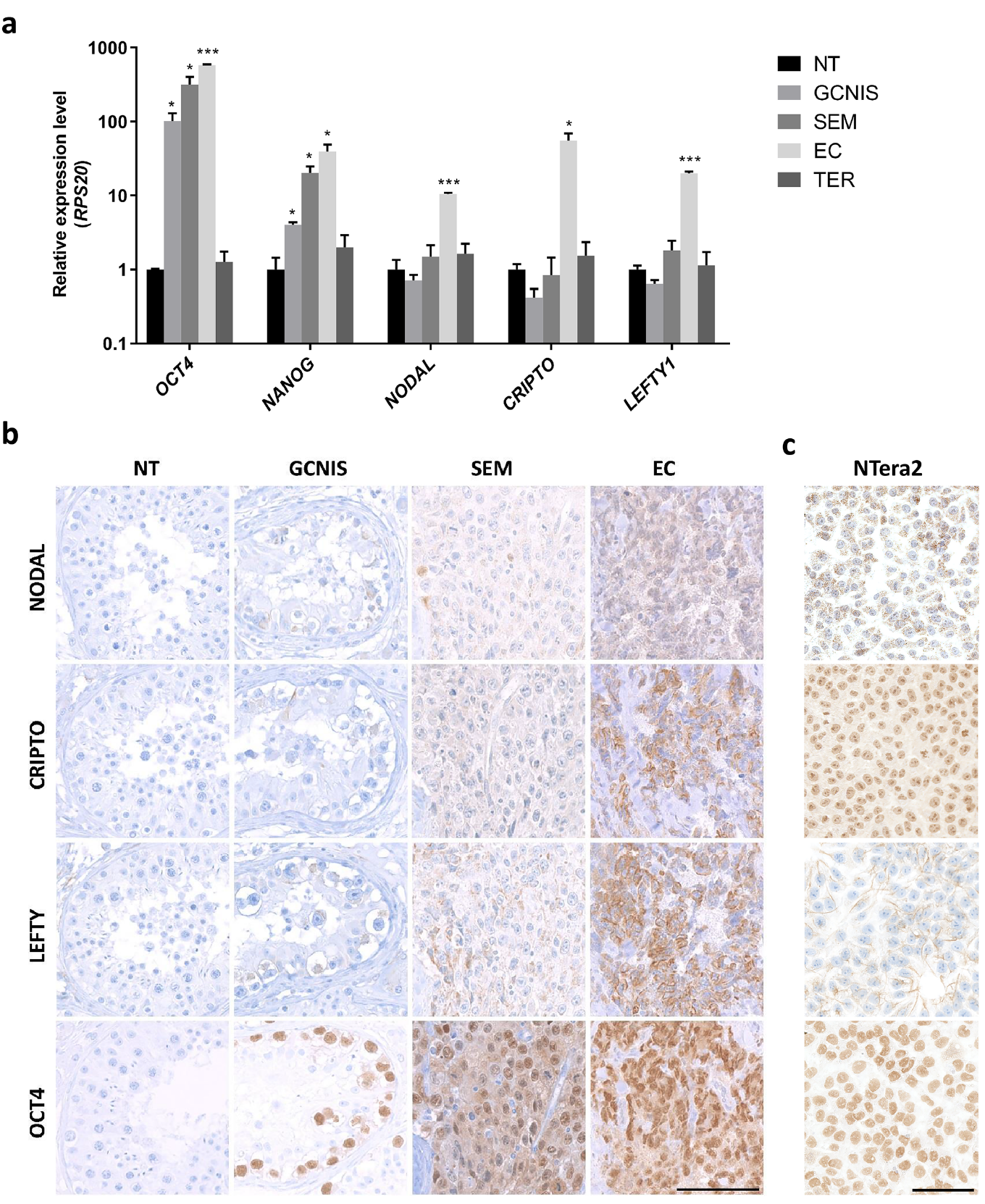


**Supplementary fig. 1. Expression of Nodal signalling factors in testis, GCNIS cells, TGCTs and TGCT-derived NTera2 cells. a)** Expression level of *OCT4*, *NANOG*, *NODAL*, *CRIPTO* and *LEFTY1* in testis tissue with complete spermatogenesis and no malignant germ cells (NT), testis samples containing GCNIS cells (GCNIS), seminoma tumour (SEM), embryonal carcinoma (EC) and teratoma (TER) examined by RT-qPCR. *RPS20* was used as reference gene. Expression level is set to 1 in NT samples. Tissue samples from eight patients were included (n=8) and measured as technical duplicates. Values represent mean ± SEM. Significant difference compared to expression in NT samples, * P<0.05, ** P<0.01 and *** P<0.001. Note logarithmic scale. **b-c)** Expression of Nodal signalling factors NODAL, CRIPTO and LEFTY (antibody detects both LEFTY1 and LEFTY2) determined by immunochemical analysis in serial sections of NT, GCNIS, SEM and EC and TGCT-derived NTera2 cells. OCT4 is included as a marker of malignant germ cells. Sections were counterstained with Mayer’s haematoxylin. Scale bars correspond to 50 μm (b) and 100 μm (c).
